# The role of phosphoinositide 3-kinase subunits in chronic thyroiditis

**DOI:** 10.1186/1756-6614-5-22

**Published:** 2012-12-21

**Authors:** Katarzyna Wojciechowska-Durczynska, Kinga Krawczyk-Rusiecka, Anna Cyniak-Magierska, Arkadiusz Zygmunt, Stanisław Sporny, Andrzej Lewinski

**Affiliations:** 1 Department of Endocrinology and Metabolic Disease, Medical University of Lodz, Polish Mother’s Memorial Hospital – Research Institute, Lodz, Poland; 2 Department of Dental Pathology, Medical University of Lodz, The Clinical-Didactic Centre, Lodz, Poland

**Keywords:** Chronic thyroiditis, PI3K isoforms

## Abstract

**Background:**

The risk of neoplastic transformation in patients with chronic thyroiditis (Hashimoto’s thyroiditis – HT) is slightly increased. Genetic background of this observation is still unclear. PI3K isoforms are linked with inflammatory and neoplastic processes, thus they appear to be interesting subjects of a research in this respect. The aim of our study was to assess the *PIK3CA*, *PIK3CB*, *PIK3CD* and *PIK3CG* genes expression levels in HT.

**Methods:**

Following conventional cytological examination, 67 thyroid FNAB specimens, received from patients with HT, were quantitatively evaluated regarding *PIK3CA*, *PIK3CB*, *PIK3CD* and *PIK3CG* expression levels by real-time PCR in the ABI PRISM ^®^7500 Sequence Detection System.

**Results:**

The performed analysis has revealed significantly higher expression levels (RQ) of *PIK3CD*, *PIK3CG* and *PIK3CA* genes in comparison with *PIK3CB* gene (p<0.05) and significantly higher gene expression level of *PIK3CD* in comparison with *PIK3CA* (p<0.05).

**Conclusion:**

The observed increased *PIK3CD*, *PIK3CG* genes expression in HT is probably related to lymphocyte infiltration commonly seen in this condition, however, the role of increased *PIK3CA* gene expression in the multi-step carcinogenesis process cannot be excluded.

## Background

Inflammatory processes protect the body against infection and injury, however, if not satisfactorily regulated, it can lead to destructive consequences, including the development of autoimmune diseases. The inflammation has also been proved to play an important role in other diseases which have not previously been considered to possess inflammatory etiology, e.g. such as cancer. Likewise, a number of researchers reported on an association between chronic thyroiditis (Hashimoto’s thyroiditis – HT) and a risk of thyroid neoplastic transformation.

The PI3K (phosphoinositide 3-kinase) signaling pathway regulates numerous cell functions in both normal and pathological states. Class I PI3K consists of four isofoms of the catalytic subunits, p110α, -β, -δ, -γ, coded by *PIK3CA*, -*B*, -*D* and –G genes, respectively. Under basal conditions, the expression of the PI3K isoforms seems to depend on transcriptional control, with mRNA levels correlating well with protein levels [1]. Moreover, it is well documented that these isoforms show different tissue distribution, i.e. PIK3CA and PIK3CB are expressed ubiquitously, while PIK3CD, PIK3CG are expressed predominantly in leucocytes [2].

Considering thyroid neoplasms, the increased activation of PI3K pathway could be related to mutations and/or amplification of *PIK3CA* gene and amplification of *PIK3CB* gene [3]. Additionally, several reports demonstrate that PIK3CD and PIK3CG subunits are essential in regulating chemokine production by leukocytes, as well as directional migration of these cells during the inflammatory process [2,3]. Thus, a role of PI3K isoforms in the thyroid immune response linked with inflammation and cancer appears to be an interesting subject of a research.

The aim of our study was to assess the *PIK3CA*, *PIK3CB*, *PIK3CD* and *PIK3CG* genes expression levels in HT.

## Material and methods

The study was approved by the Ethics Committee of the Medical University of Lodz, Poland. Written consent was obtained from all the patients subjected to the fine needle aspiration biopsy (FNAB).

Cytological specimens from 67 patients (64 women, 3 men) with chronic thyroiditis were analyzed. Following preparation of smears for cytological examination, extraction of total RNA from remnant biopsy needle material was obtained by an RNeasy Micro Kit (Qiagen), using modified Chomczynski and Sacchi’s method. The purity of total RNA was assessed by NanoDrop^®^ ND-100 spectrophotometr. One hundred nanogram of total RNA was used in the first strand cDNA synthesis with TaqMan^®^ Reverse Transcription Reagents (Applied Biosystems), according to manufacturers’ instruction.

Real-time PCR was performed on the ABI PRISM^®^ 7500 Sequence Detection System (Applied Biosystems) by using *Taq*Man^®^ Universal PCR Master Mix (Applied Biosystems) and *Taq*Man^®^ Gene Expression Assays probe and primer mix (Applied Biosystems) according to the manufacturers’ specification. The Assay Identification numbers were: *PIK3CA* - Hs00180679_m1; *PIK3CB* - Hs00178872_m1; *PIK3CD* - Hs00192399_m1; *PIK3CG* - Hs00176916_m1. Thermal cycler conditions were as follows: hold for 10 min. at 95°C, followed by two-step PCR for 50 cycles of 95°C for 15 s and followed by 60°C for 1 min. Amplification reactions were performed in triplicate for each sample, and the results were normalized to the *ACTB* gene expression level (*ACTB* Assay Identification number: Hs99999903_m1). Macroscopically unchanged thyroid tissue, surgically removed from patients with nodular goitre, served as a control for *real*-*time* PCR experiment.

An analysis of relative gene expression data was performed, using the 2^-ΔΔCT^ method on an ABI PRISM^®^ 7500 Sequence Detection System Software (Applied Biosystems). The fold change in studied gene expression, normalised to endogenous control, was calculated using formula: RQ = 2^-ΔΔCT^.

In order to compare the relative expression (RQ) of *PIK3CA*, *PIK3CB*, *PIK3CD* and *PIK3CG* genes in HT, the data were statistically analysed using Kruskal-Wallis’ and Newman-Keuls’ tests. In all analyses, statistical significance was considered achieved at a value of p<0.05.

## Results

**Figure 1:**
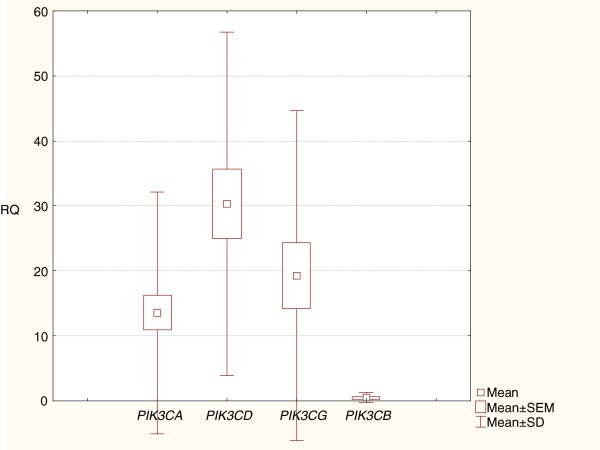
**Box-and-whisker plots representing the expression of *PIK3CA*, *PIK3CB*, *PIK3CD *and *PIK3CG *genes in HT. **The results are calculated as RQ values. Whiskers represent standard deviation for particular genes. Boxes represent standard error of mean. Small boxes illustrate means. The results were statistically analyzed, using Kruskal-Wallis’ and Newman-Keuls’ tests (p<0.05).

**Figure 2:**
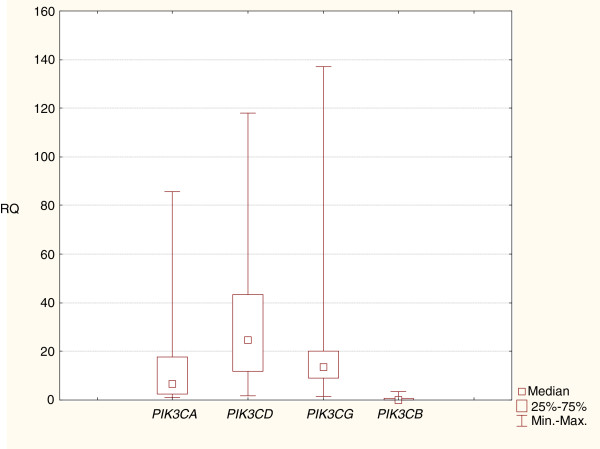
**Box-and-whisker plots representing the expression of *PIK3CA*, *PIK3CB*, *PIK3CD *and *PIK3CG *genes in HT. **The results are calculated as RQ values. Whiskers represent minimum and maximum value for particular genes. Boxes represent lower quartile and upper quartile of values. Small boxes illustrate median values. The results were statistically analyzed, using Kruskal-Wallis’ and Newman-Keuls’ tests (p<0.05).

The performed analysis has revealed significantly higher expression levels (RQ) of *PIK3CD*, *PIK3CG* and *PIK3CA* genes in comparison with *PIK3CB* gene (p<0.05). Further statistical analysis proved significantly higher gene expression level of *PIK3CD* in comparison with *PIK3CA* (p<0.05) (Figure 1, 2). 

## Discussion

Chronic thyroiditis affects a significant portion of endocrinologic patients, with considerable quality-of-life burden. When discussing chronic thyroiditis, it is worth stressing that former studies revealed infiltration of thyroid tissue by a mixed population of T and B lymphocytes [4]. Moreover, chronic thyroiditis is characterized by the presence of thyroid antibodies; antibodies against thyroperoxidase (TPO) are the most specific ones.

PI3K isoforms control inflammation processes at many levels, from the generation of inflammatory cells to their migration and function. However, the precise role of PI3K in the regulation of immune process in the thyroid remains open to refinement. Genetic approaches have been employed to assess the contribution of class I PI3K isoforms in thyroid immune process. Our present findings have confirmed increased expression of *PIK3CA*, *PIK3CD* and *PIK3CG* genes and decreased expression of *PIK3CB* gene in HT. Further statistical analysis has revealed the increased expression level of *PIK3CD* in comparison with *PIK3CA*.

Our results are partially in agreement with previous observations that PIK3CD subunit is expressed at high levels in lymphocytes and lymphoid tissue and may – therefore - play a role in PI3K mediated signaling in the immune system [5]. In addition, PIK3CD has been implicated in leukocyte endothelial transmigration during inflammation [6] but it is not clear whether this effect is caused by PIK3CD expression in leukocytes themselves, in endothelial cells or by both.

The role of PIK3CD isoform in thyroid carcinogenesis remains to be determined, however it is worth stressing that PIK3CD contributes in other neoplastic processes. In acute myeloid leukemia cells, PIK3CD is the only class I PI3K isoform that consistently is detected [7,8]. At the same time, PIK3CD is expressed to moderate degree in certain neoplastic cells of non-leukocyte origin, such as melanoma cells, breast and colon cancer cells. However, large discrepancies in expression levels in cell lines, even of the same tissue origin, were noted [9].

The explanation of our findings can also be related to the process of antibodies generation which requires collaboration between B and T cells within germinal centers, the process in which PIK3CD subunit is essential in T cells just for the germinal centre reaction. Additionally, it has also been found that formation of T follicular helper cells is critically dependent on PIK3CD [10].

Furthermore, the contribution of PIK3CD subunit in other autoimmune disorders, like rheumatoid arthritis has also been confirmed and PIK3CD is a promising therapeutic target in this disease because of its role to leukocyte biology [11]. It has also been demonstrated that PIK3CD is a major regulator of PDGF-mediated fibroblast growth [11], the observation which can support the role of PIK3CD also in the process of thyroid fibrosis in HT.

In our present investigation we have demonstrated high expression of *PIK3CG* gene in HT. It has previously been regarded that PIK3CG subunit is required to allow chemotactic migration of neutrophils, macrophages, and effector CD8 T cells to inflammatory sites [12,13]. The increased *PIK3CG* gene copy number has been documented in ovarian cancer, as well [14].

Previous findings by Ghigo et al. [15], together with our present observations, suggest that PIK3CD and PIKCG can be involved in inflammation processes and can influence the immune system. Both PIK3CD and PIK3CG had also been identified as validated drug targets in immune and inflammatory diseases [16]. Nevertheless, the non-redundant but related roles of PIK3CD and PIK3CG have made it difficult to determine which of the two isoforms (alone or in combination) is best targeted in a particular inflammatory disorder.

However, it should be recalled that the increased expression of p110 isoform, especially of isoform of p110α, induced also oncogenic transformation [3]. Amplification and mutations in *PIK3CA* gene have been reported in many human cancers, including thyroid cancer, especially in follicular thyroid carcinoma and undifferentiated thyroid carcinoma [3]. Still, little is known about the precise role of *PIK3CA* gene in inflammatory disorders in thyroid gland, however, the increased expression in Riedel’s thyroiditis has previously been observed in our laboratory [17].

In conclusion, the present study speaks for contribution of PIK3CD, PIK3CG and PIK3CA isoforms to the autoimmune processes in the thyroid. The observed increased *PIK3CD*, *PIK3CG* genes expression in HT is probably related to infiltration of lymphocytes, however, the role of the increased *PIK3CA* gene expression in the multi-step carcinogenesis process cannot be excluded, either.

## Competing interests

The authors declare that they have no competing interests.

## Authors’ contributions

KW-D participated in a design of the study and also she carried out molecular genetic procedures and prepared the draft of a manuscript. KK-R and AC-M both participated in performing molecular studies. AZ participated in data acquisition and in coordination of the study. SS assessed the thyroid cytological specimens. AL senior author, designed the study and wrote the final version of manuscript. All authors have read and approved the final manuscript.
